# An Expert System for COVID-19 Infection Tracking in Lungs Using Image Processing and Deep Learning Techniques

**DOI:** 10.1155/2021/1896762

**Published:** 2021-11-13

**Authors:** Umashankar Subramaniam, M. Monica Subashini, Dhafer Almakhles, Alagar Karthick, S. Manoharan

**Affiliations:** ^1^Department of Communications and Networks, Prince Sultan University, 11586 Riyadh, Saudi Arabia; ^2^Department of Control and Automation, School of Electrical Engineering, Vellore Institute of Technology, 632014 Vellore, India; ^3^Renewable Energy Lab, Department of Electrical and Electronics Engineering, KPR Institute of Engineering and Technology, Arasur, Coimbatore 641407, Tamilnadu, India; ^4^Department of Computer Science, School of Informatics and Electrical Engineering, Institute of Technology, Ambo University, Ambo Post Box No.: 19, Ethiopia

## Abstract

The proposed method introduces algorithms for the preprocessing of normal, COVID-19, and pneumonia X-ray lung images which promote the accuracy of classification when compared with raw (unprocessed) X-ray lung images. Preprocessing of an image improves the quality of an image increasing the intersection over union scores in segmentation of lungs from the X-ray images. The authors have implemented an efficient preprocessing and classification technique for respiratory disease detection. In this proposed method, the histogram of oriented gradients (HOG) algorithm, Haar transform (Haar), and local binary pattern (LBP) algorithm were applied on lung X-ray images to extract the best features and segment the left lung and right lung. The segmentation of lungs from the X-ray can improve the accuracy of results in COVID-19 detection algorithms or any machine/deep learning techniques. The segmented lungs are validated over intersection over union scores to compare the algorithms. The preprocessed X-ray image results in better accuracy in classification for all three classes (normal/COVID-19/pneumonia) than unprocessed raw images. VGGNet, AlexNet, Resnet, and the proposed deep neural network were implemented for the classification of respiratory diseases. Among these architectures, the proposed deep neural network outperformed the other models with better classification accuracy.

## 1. Introduction

Emerging pathogens are a big concern for global public health, and technology may help classify potential cases more rapidly in order to bring in timely treatments [1, 2]. The Envision 2030 agenda of the United Nations have included 17 sustainable development goals towards a promising future for persons with disabilities aligned with Saudi Vision 2030. As per the SD goals set and implemented by the United Nations, the proposed work targets promoting the transformation of disabilities. This work promises good health and well-being (SDG 3) by diagnosing respiratory diseases at the earlier stages based on chest X-ray images. To achieve this, the authors have utilized innovation techniques, infrastructure (SDG 9), and international partnerships (SDG 17) to transform the world into a better place to live.

The novel coronavirus identified in December resulted in significant quarantines worldwide, including large cities, villages, and public areas [3]. The huge impact of COVID-19 is due to a lack of testing and medical errors. COVID-19 is a disease that mainly affects the lungs [4] apart from pneumonia. This can exhibit various patterns or pathological symptoms in the lungs based on different causes, and no particular symptom can indicate its severe impact on the lungs. Hence, the diagnosis should be initiated at an early stage. Based on the symptoms, when a person approaches a clinic, few tests are done for confirmation of the disease along with X-ray images. X-ray images help in analyzing the condition of the lung and the stage of the disease. Preprocessing of these images is absolutely necessary for accurate diagnosis.

## 2. Contributions and Limitations

This paper focuses on image preprocessing techniques applied on pneumonia, COVID-19 dataset (X-ray images), and diagnosis of diseases from the processed X-ray images

To diagnose the disease using X-ray images, different CNN architectures were implemented and compared to find out the best architecture

In this study, deep neural network architecture is proposed to diagnose normal/pneumonia/COVID-19 or bacterial infected disease

This model is trained on COVID-19 X-ray images, pneumonia X-ray images, and normal lung X-ray images

The failure to detect the early stages of COVID-19 is one of the most significant limitations of chest radiography research

But well-trained deep learning models can concentrate on points that the human eye cannot notice, and this method was successful in achieving the same

The only limitation to this method is the availability of datasets (training/testing) on normal/COVID-19/pneumonia X-ray images. The literature survey enabled the use of resources in fetching the dataset from various medical repositories

## 3. Related Work

COVID-19 has to be detected properly without any negligence else can lead to a severe impact on the country's economy and country's citizen health [5]. The person who is suspicious of COVID-19 is suggested to undergo a chest X-ray. Analysis of X-rays by humans can lead to various human errors, which can lead to a huge impact on patients and society. So, a computer-aided system can help the doctors for proper analysis of lungs of the COVID-19 affected human. Throughout underdeveloped and developing nations, where the number of patients is high and medical care cannot be adequately delivered, these programs may be a tremendous benefit [6, 7]. The authors have worked on X-ray imaging techniques for the detection of bone fractures. They have applied edge detection and segmentation techniques to ease the process of the diagnosis system. These methods will reduce the processing time and other physical evaluation procedures [8]. So, while working with X-ray images, we need to consider the noises which have to be reduced. The random noises occurring during the process of image acquisition degrades the image quality leading to an incorrect diagnosis. Researchers suggest the application of the temporal recursive filter. Also, they propose an improved self-adaptive filter. This was a combination of FPGA with image processing techniques [9]. The authors have recommended region localization which offers a close level of precision. Few other image preprocessing techniques are adaptive histogram-based equalization, adaptive contrast enhancement, and histogram equalization. There is the presence of multiple noises during capturing the images because of device mobility and motion artefact [10]. But in X-ray images mostly Gaussian, salt and pepper noises are present. To reduce the noise, a digital median filtering technique is used as per the researches.

Chest X-rays aid in the diagnosis of pneumonia. Researchers seek the help of CNN in classifying normal and abnormal X-rays [11]. The feature extracted from the lung X-ray improves the functionality of the classifier. This method is useful where a large dataset is received. In another similar work, deep learning techniques are applied for the analysis of chest X-rays. Pulmonary infections are easily identified using these radiography images. This is extended in the detection of coronavirus disease [12]. The authors have brought hope in applying artificial intelligence in the early detection of the disease. Supervised learning techniques have been applied in the classification of a normal/abnormal pneumonia dataset. A labelled dataset aids this process in reducing the error [13]. CNN has been trained with nonaugmented data. The researchers have suggested a novel deep learning architecture for pneumonia detection [14]. They have applied transfer learning for efficient classification. The features were extracted and pretrained on ImageNet. A classifier predicted the possibility of pneumonia. The developed ensemble model provided an accuracy of 96.4%. They have utilized the Guangzhou Women and Children's Medical Center dataset. Ozturk et al. [15] proposed a model to provide diagnostics on normal, COVID-19, and pneumonia with binary and multiclass classification. Their model provided classification accuracy of 98.08% for binary classes and 87.02% for multiclass cases. In a recent research, the authors have implemented a deep neural network to differentiate COVID-19-induced pneumonia and other virus-induced pneumonia from chest X-ray images [16]. Researches have analyzed the effectiveness of the deep learning model VGG16 for the detection of COVID-19 and pneumonia [17]. The authors have claimed the work as a screening test based on the sensitivity and degree of specificity. In a similar study [18], the authors have presented an automatic COVID-19 screening system which used radiomic texture descriptors to differentiate the CXR images to identify the normal, COVID-19, and suspected infected patients. Researchers have predicted the severity of COVID-19 from chest X-ray images [19]. Their method can measure lung infections as well as monitor treatment in ICU. Sharma et al. [20] have applied transfer learning for the classification of respiratory diseases including tuberculosis, pneumonia, COVID-19, and normal lung. Hence, based on the recent literature [21–29], the proposed method has implemented few efficient image preprocessing algorithms and deep learning models to diagnose respiratory diseases. The deep learning models developed is efficient when the input images are preprocessed with contrast enhancement, segmentation, and feature extraction. The accuracy improved in the classification of respiratory diseases.

## 4. Methodology

**Figure 1:**
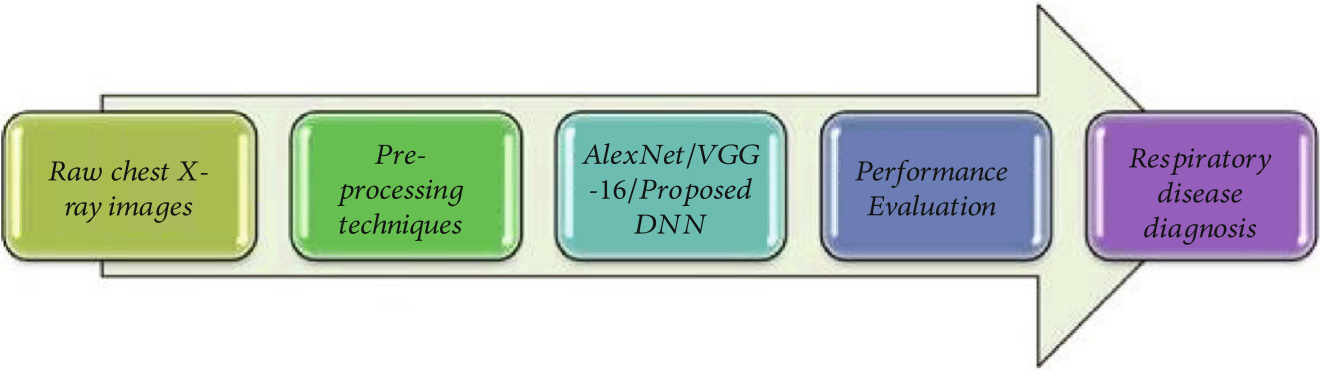
Process flow.

The proposed method involves preprocessing and classification of lung X-ray images using image processing algorithms and deep learning techniques as shown in Figure 1.

The X-ray images are preprocessed to locate the lungs. As per the literature, the stages of COVID-19 and pneumonia can be detected from the lung. Hence, the left lung and right lung have to be thoroughly analyzed to differentiate normal/abnormal images. Various algorithms on preprocessing have been applied, and the performance is evaluated. The processed data would be used as labelled data for the convolutional neural network model for the classification of respiratory diseases.

## 5. Dataset

**Figure 2:**
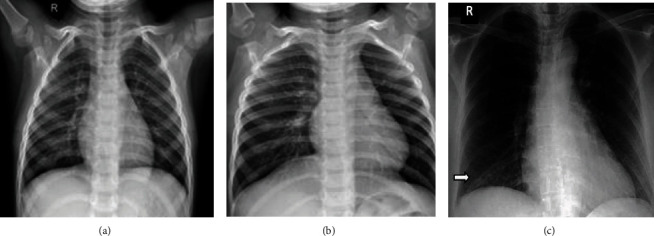
Sample image from training dataset: (a) normal; (b) pneumonia; (c) COVID-19.

**Figure 3:**
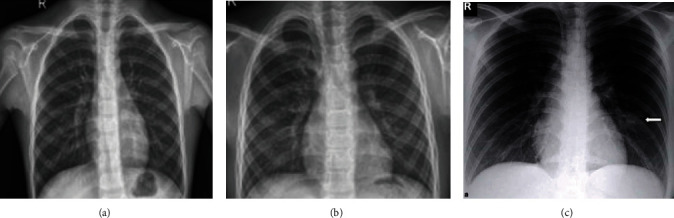
Sample image from testing dataset: (a) normal; (b) pneumonia; (c) COVID-19.

**Figure 4:**
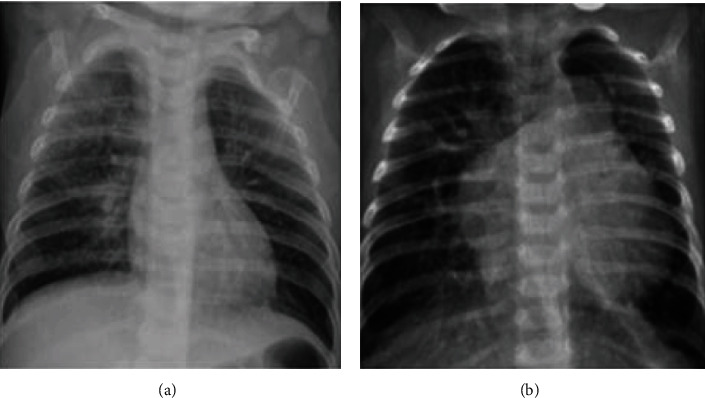
Sample image from validated dataset: (a) normal; (b) pneumonia.

Data is the first step in establishing any method or therapy for the diagnosis. The X-ray images of a COVID-19/pneumonia patient's lung are used for preprocessing. The data is collected from a public database consisting of X-ray and CT scan images for various diseases, a project approved by the University of Montreal's Ethics Committee. The images of COVID-19 are drawn out from a public database and used for further processing. 78% of COVID-19 X-ray images are randomly split into the training dataset for training the classifier, and 22% of COVID-19 X-ray images are used for validation. The lung part in images of the training dataset is segmented physically to train the machine learning model to segment the lungs from the COVID-19 lung X-rays. Similarly, a total of 1200 images including normal and pneumonia (training and testing) were utilized for the proposed method and shown in Table 1. Also, the sample dataset is shown in Figures 2–4.

## 6. The Proposed Model Development for Respiratory Disease Detection

**Figure 5:**
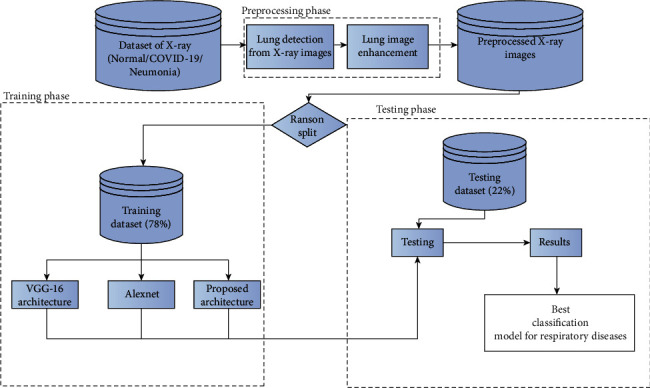
Model development.

The following steps have been followed in the model development and depicted in Figure 5:

- Apply deep learning edge detection model on COVID-19 images
- Apply deep learning edge detection model on pneumonia images
- Apply deep learning edge detection model on normal images
- Make COVID-19 directory which contains training (110 images) and testing (30 images) directories
- Make pneumonia directory which contains training (110 images) and testing (30 images) directories
- Make a directory which contains normal training (110 images) and testing (30 images) directories
- Develop a CNN model using pretrained model
- Develop architecture for CNN model
- Develop a hybrid model with machine learning and deep learning

## 7. Preprocessing Techniques

### 7.1. Haar Cascade Classifier

Lung detection using Haar feature-based cascade classifiers is an effective location technique proposed by Paul Viola and Michael Jones in 2001. It is an AI-based methodology where a cascade work is prepared from a great deal of positive and negative pictures. It is then used to distinguish questions in different pictures.

In this proposed method, the discovery of lungs in X-ray images is defined. At first, the algorithm needs positive (pictures of appearances) and negative (pictures without lungs) to prepare the classifier followed by feature extraction from the images to train the model. The obtained Haar features are utilized. The features are mostly the same as the convolutional kernel. Each element is a solitary worth acquired by deducting the aggregate of pixels under a white rectangle shape from the entirety of pixels under the dark rectangle shape.

The cascading of the classifiers helps to evaluate even the subimages with the greatest likelihood for all Haar transform that differentiates an entity. This also helps in adjusting a classifier's accuracy. Viola and Jones were able to detect a human face with an accuracy rate of 95 per cent using only 200 simple features. Hence, the Haar cascade classifier is trained to classify lungs using the extracted features. This gentle AdaBoost algorithm and Haar feature algorithms have been implemented to train these classifiers, and in this process, more than 6000 features were included to classify with greater accuracy.

### 7.2. HOG for Lung Segmentation

HOG, or histogram of oriented gradients, is an element descriptor that is frequently used to extricate features from picture information. It is broadly utilized in PC vision errands for object location. We should take a gander at some significant parts of HOG that make it unique in relation to other component descriptors.

The HOG descriptor centers on the structure or the state of an item. Hoard can give the edge course too. This is finished by extracting the slope and direction of the edges. Furthermore, these directions are determined in “restricted” parcels. This implies that the total picture is separated into littler locales, and for every area, the inclinations and direction are determined. The HOG would produce a histogram for every one of these locales independently. The histograms are made utilizing the slopes and directions of the pixel esteems, thus the name “histogram of oriented gradients”

- Preprocess the data (64 × 128)
- Calculating gradients (directions *x* and *y*)
- Calculate the magnitude and orientation

Using each pixel's magnitude and orientation in the X-ray, it is graded into a lung image or not. Support vector machine (SVM) helps in classifying an image and works more efficiently with HOG transform. The SVM model is essentially a reflection in multidimensional space of various classes in a hyperplane. The hyperplane is created by SVM iteratively to minimize the cost function. SVM is aimed at segmenting the datasets into classes in order to find a maximal absolute hyperplane.

### 7.3. LBP Transform for Lung Segmentation

The substance of an individual passes on huge information of data about the lung and enthusiastic condition of the individual. The simplest operator is to set the middle pixel “Pm” value as a threshold and associate it with the pixels in the neighbourhood, Pn. The value of Pm is calculated. All threshold values of neighbourhood points are multiplied by the corresponding proportion and added. The LBP code for *k*-bit can be calculated as

(1) $$\begin{array}{c} \text{LBP}=\overset{i=k-1}{\underset{i=0}{\sum }}F\left(\text{Pn}-\text{Pm}\right)2^{i}. \end{array}$$

Pn represents the *n*^th^ neighbouring pixel, and “*m*” represents the center pixel. The histogram features of size 2^*i*^ are extracted from the obtained local binary pattern code.

The lung region is first partitioned into little areas from which local binary patterns (LBP) and histograms are extricated and linked into a solitary component vector. This element vector shapes a proficient portrayal of the lung and is utilized to quantify likenesses between pictures.

**Figure 6:**
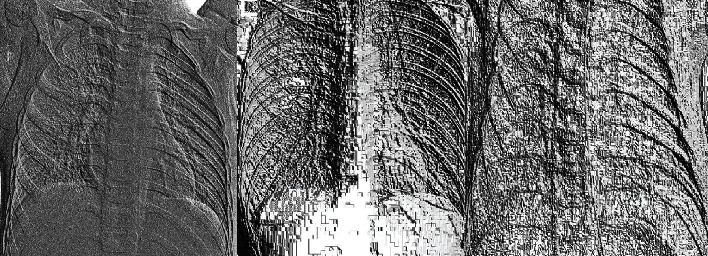
Feature descripted images: (a) normal, (b) pneumonia, and (c) COVID-19.

The LBP transformed image has features that can be used for classification, and sample images are shown in Figures 6(a)–6(c). Feature extraction helps the classification model in classifying the image with better accuracy.

### 7.4. Diagnosis of Disease

**Figure 7:**
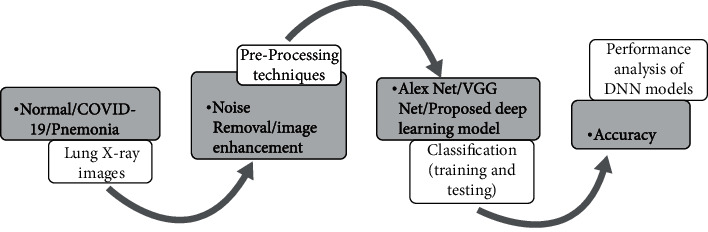
Block diagram on diagnosing the disease.

The dataset consists of normal lung images, bacterial pneumonia affected lung images, and COVID-19 affected lung images. The images are preprocessed to remove noise and to enhance the image quality. The images are scaled and cropped for increasing the performance of the proposed deep learning model. After preprocessing, AlexNet, VGGNet, and the proposed neural network are trained on normal/abnormal lung images. After training, the models are tested with data and the algorithm with the best accuracy is selected for further diagnosing the process. The process is represented in the block diagram in Figure 7.

### 7.5. Implementation

#### 7.5.1. Preprocessing Algorithms

**Figure 8:**
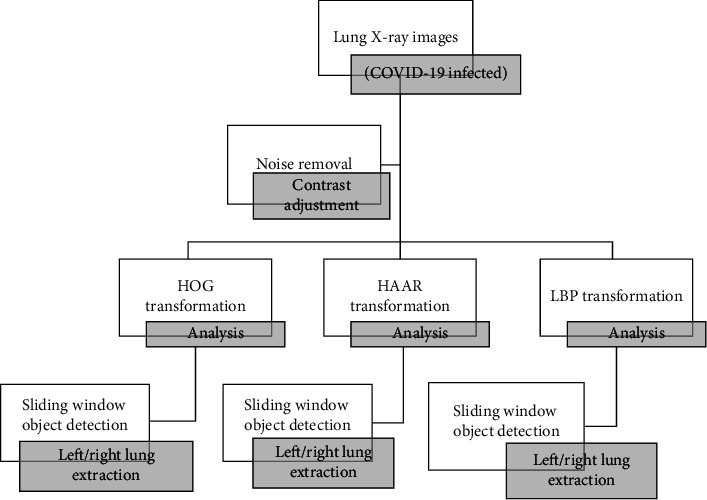
Preprocessing of the X-ray images.

**Figure 9:**
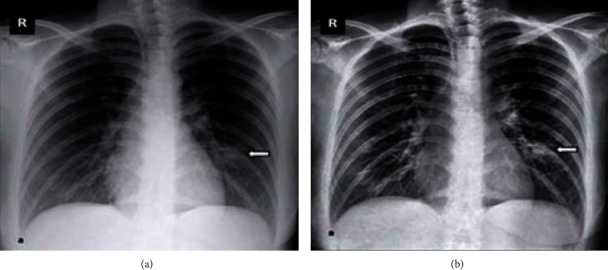
(a) Left image shows normal lung image; (b) right image shows processed image using contrast adjustment.

COVID-19 lung images are used to train the classifier in the detection of lungs from the X-ray images. Various transformations like HOG, Haar, and LBP were applied on images, and depending on the accurate segmentation of lungs, the best accurate algorithm for the detection has been chosen. In Figure 8, the process is depicted on preprocessing of COVID-19 images, and extraction of the lungs using the sliding window technique is highlighted. The preprocessed images (contrast enhancement) are shown in Figure 9.

#### 7.5.2. Noise Removal

**Figure 10:**
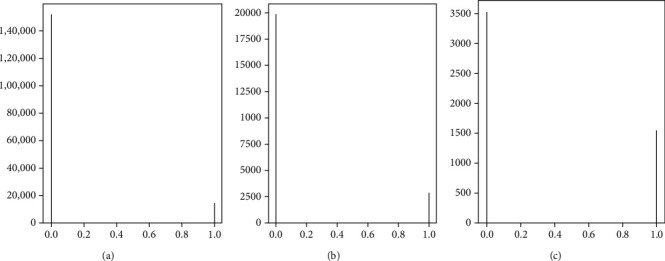
(a) Normal; (b) pneumonia; (c) COVID-19.

The noises from the dataset are removed to get the best features from the COVID-19/pneumonia lung images. Some of the preprocessing techniques that can be applied to images are contrast adjustment, intensity adjustment, histogram equalization, binarization, morphology operation, etc. For lung X-ray images, contrast adjustment and histogram equalization perform better when compared with other preprocessing techniques. Contrast adjustment of a COVID-19 lung image has produced a better image to extract the features from the lungs. These features can further be used for the detection of lungs from X-ray images using various lung detection algorithms. As shown in Figures 10(a)–10(c), histogram equalized images represent normal, pneumonia, and COVID-19 with gray level and frequency count. The histogram equalization on an image produces images with better quality without loss of information. This contrast adjustment using histogram equalization helps in extracting better features in lung detection algorithms.

#### 7.5.3. Detection of Lungs Using HOG Feature Vector, Haar Transform, and LBP Transform

The algorithm used for lung detection is the sliding window technique where a window of size starting from 2 × 2 × 3 to *N* × *N* × 3 is used to detect the left and right lungs of an image. The window of constant size is allowed to slide over the entire image, and the window will be utilized with transform techniques to locate lungs. The algorithm shown below can be used in various applications to get better accuracy.

Step 1. Contrast adjustment of X-ray image.

Step 2. Sliding window with window size from 2 to size of the image (*N*).

Step 3. Classification of image in window into left the lung using HOG feature descriptor, Haar transform, and LBP transform.

Step 4. Classification of image in window into right lung using HOG feature descriptor, Haar transform, and LBP transform.

Step 5. Repeat steps from Steps 2 to 4 until the left lung and right lungs are extracted properly with every algorithm.

Step 6. Comparing the extracted images of lungs to predict the best algorithm for feature description.

## 8. Proposed Deep Neural Network

The proposed DNN model is a powerful network which is capable of getting higher accuracy on classification of respiratory diseases. This architecture is used to classify X-ray images of lungs into COVID-19, pneumonia caused by bacteria, and a normal lung image. This architecture can also be extended to various object classification models.

In medical image analysis, accuracy is the major factor for architecture to be implemented in any system. This model consists of 7 sequentially connected convolutional layers with different size filters. These convolutional layers are sequentially connected to three fully connected layers. The major characteristics of this architecture are features of the image passed through strided convolutions and max pooling layers. The architecture has used 5 × 5 and 3 × 3 size filters. The architecture has convolutions, max pooling, dropout, and ReLu activation layers.

### 8.1. Architecture

The proposed architecture has 10 layers where 7 layers are convolutional layers and the remaining 3 are fully connected layers and shown in Table 2. A fixed size of 448 × 448 × 3 RGB lung image of COVID-19 should be given as input to the network. After preprocessing of the image, the image has been subjected to the network for classification. The first layer in architecture uses “5 × 5” size filter with stride 1 pixel. All other layers in architecture use “3 × 3” size filter with a stride of 1 pixel. These filters can help to get all features of an image. Max pooling is performed on different layers over 2 × 2 size filter with stride 1. Each convolutional layer is followed by the Rectified Linear Unit (ReLu) to add nonlinearity to the model. This nonlinearity can classify the images with high accuracy. The architecture contains 3 fully connected dense layers where the first layer is of size “4096” and the second layer is of size “1024.” The final layer which classifies the image is of size “3”. Softmax function is applied to the output layer to classify the image.

## 9. AlexNet Deep Neural Network

AlexNet is an incredibly adaptable model which, on particularly problematic datasets, can achieve high accuracy. Regardless, dispensing with the whole of the traditional layers will profoundly degenerate AlexNet's introduction. AlexNet is a fundamental plan for any movement and can have enormous consequences for issues with automated thinking, keeping watch for PC vision. The model includes five progressively associated convolutional layers of lessening channel size, followed by three totally associated layers. One of AlexNet's fundamental features is the incredibly speedy down testing of the widely appealing depictions through strided convolutions and max pooling layers. The delayed consequences of AlexNet (Table 3) have demonstrated that on an uncommonly testing dataset, an immense significant convolutionary neural association is good for achieving record-breaking results using coordinated learning. AlexNet is made out of eight layers. The underlying 5 are convolutionary, and the last 3 are completely related layers. We also have couple “layers,” called pooling and order.

## 10. VGGNet

VGG is a contraction for the Oxford University Visual Geometric Group, and VGG-16 is a 16-layer network proposed by the Visual Geometric Group. These 16 layers contain likely limits and various layers; for instance, the max pool layer is available (Table 4), yet no workable limits are joined.

### 10.1. Architecture

The plan is essential, followed by a most extreme pooling layer of two flanking squares of 2 convolution layers; by then, it has three adjoining squares of 3 convolution layers, followed by max pooling; finally, we have three thick layers. The last 3 convolution layers have obvious profundities in different models. The fundamental examination is that after each most extreme pooling measure, the size is reduced considerably. A fixed size (224∗224) RGB picture is given as a commitment to this association which infers that the structure has been made (224, 224, 3). The primary preplanning is that they deducted the mean RGB regard from each pixel, assessed over the whole getting ready grouping. Pieces with a size of 3∗3 and a phase size of 1 pixel were used to cover the entire idea of the image. With stage 2, over 2∗2 pixel windows, max pooling is accomplished. Three totally related layers were progressively connected to these convolutionary layers, the underlying two of which were 4096 in size, and afterward, the last layer was a layer with 1000 channels for 1000-way ILSVRC gathering and a softmax feature.

## 11. Results and Discussion

### 11.1. Extracting Features from Haar Transform

**Figure 11:**
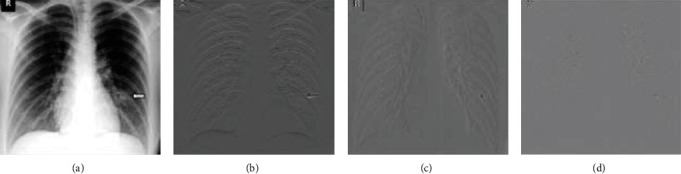
(a) Original image; (b) horizontal detail; (c) vertical detail; (d) diagonal detail.

The original image in Figure 11(a) is taken, and various Haar transform filters are applied to obtain different image characteristics. As shown in Figures 11(a)–11(d), few features are extracted from the original X-ray image in horizontal, vertical, and diagonal, and the respective feature image is created. These characteristics can be extracted using various kernels. This process with several thousand features extracted from images provides better accuracy.

### 11.2. HOG Feature Descriptor

**Figure 12:**
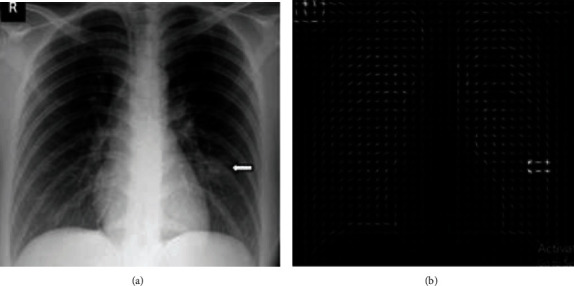
(a) Original image; (b) histogram of oriented gradients.

For each pixel, the gradient direction and magnitudes are determined, and feature vectors are extracted using histograms. The vector of the extracted feature from an image helps extract the features from the image. In classifying a picture into a lung image or a nonlung image, these features are helpful. The illustration in Figure 12(a) is a pictorial representation of the magnitudes and directions of the gradient. In Figure 12(b), whenever there is no shift in contrast, hog gradients are almost zero.

### 11.3. LBP Feature Descriptor

**Figure 13:**
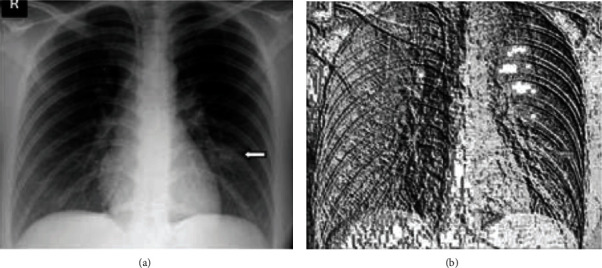
(a) Original image; (b) local binary pattern image.

The local binary pattern is a plain and gray-scale invariant feature descriptor which is highly used for extracting features for an image in a classification model. In LBP, by thresholding its neighbourhood pixels to either 0 or 1 depending on the center pixel value, a binary code is created at each pixel.  Figure 13(b) is an image which contains all the features descripted from the original COVID-19 lung image in Figure 13(a).

**Figure 14:**
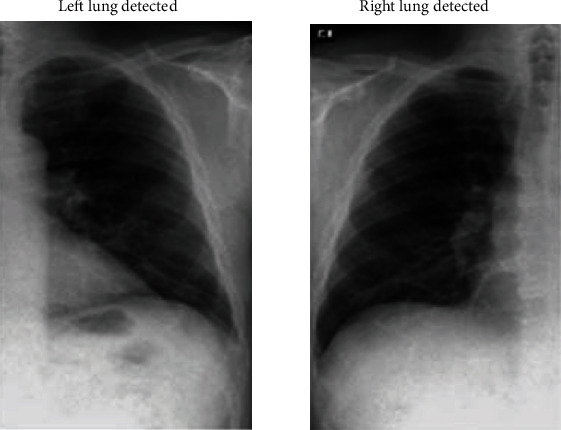
Left and right lung detection using Haar transform.

**Figure 15:**
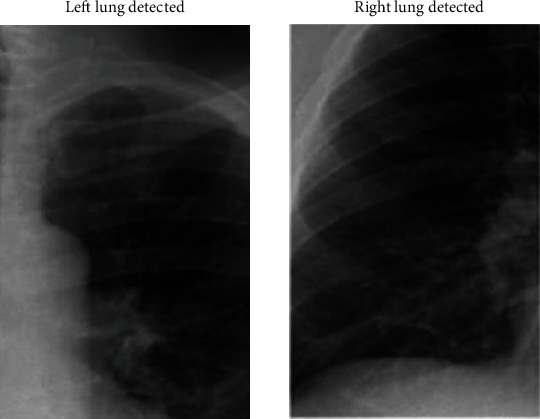
Left and right lung detection using local binary pattern algorithm.

In Figure 14, the left and right lungs are detected after extracting thousands of features from the image in which the lungs have to be detected. Cascade classification is used to classify the image into lung or not. The cascade classifier uses the features extracted using Haar transform to get the best accuracy in the classification process. In Figure 15, the lungs are detected using the feature description vector of every pixel in the original COVID-19 image. Through the support vector machine algorithm, the image would be subjected to identification of lungs. The support vector machine helps in classifying an image with better accuracy. The LBP transform helps in extracting features at every pixel with the help of neighbouring pixels. Various Machine Learning Algorithms can be applied to LBP transformed images to classify a COVID-19 image. The results in Figure 14 are classified using the support vector machine and provided better accuracy.

### 11.4. Result Analysis and Performance Evaluation

The objective of this proposed method is to classify lung X-ray images into normal (without any viral/bacterial infections), COVID-19 disease, and pneumonia disease using deep learning techniques. The study revealed that the preprocessing of data improved the performance of the developed model in diagnosing respiratory diseases. This study also implemented three deep learning models for the classification. AlexNet and VGG-16 were the pretrained models, and the proposed deep neural networks were the developed model. A comparative analysis of these three models revealed that the developed model outperformed the pretrained model in terms of IoU scores.

The results are promising (91%) in multiclass classification when compared to the results of existing works in respiratory system disease diagnosis and tabulated in Table 5.

To measure the best algorithm out of the above three algorithms, intersection over union scores are calculated. Intersection over union can be calculated by

(2) $$\begin{array}{c} \text{IOU}=\frac{\text{Area of overlap}}{\text{Area of union}}. \end{array}$$

In equation (2), the area of overlap is the intersection of the area of ground truth image with the area of the predicted image, and the area of union is the union of the area of ground truth image with the area of the predicted image. The algorithm with IOU scores above 0.5 can be used as the detector algorithm. As per Table 6, detection of lungs using Haar transform was performing better when compared with the other two algorithms

**Figure 16:**
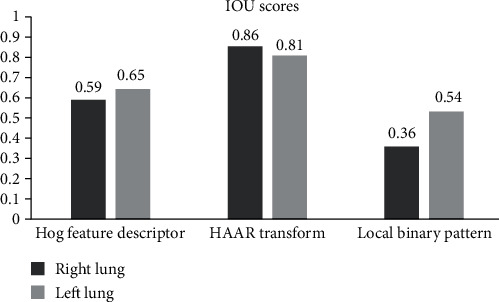
IOU scores of proposed image processing algorithms.

In Figure 16, the applied algorithms for feature extraction are plotted with their IOU scores for the left and right lungs.

The results clearly depict that Haar transform outperformed the Hog feature descriptor and local binary pattern methods.

### 11.5. Evaluation of Classification Models

**Figure 17:**
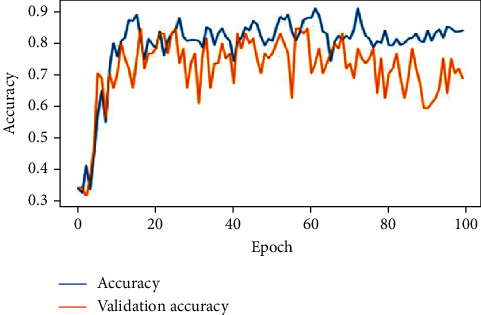
VGGNet after 100 epochs.

**Figure 18:**
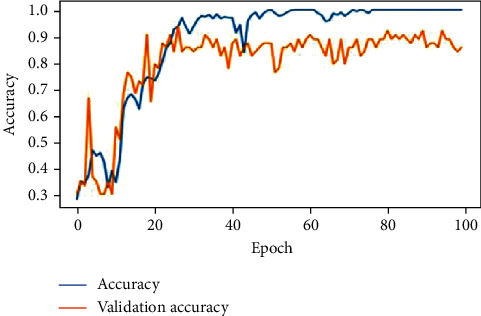
AlexNet after 100 epochs.

**Figure 19:**
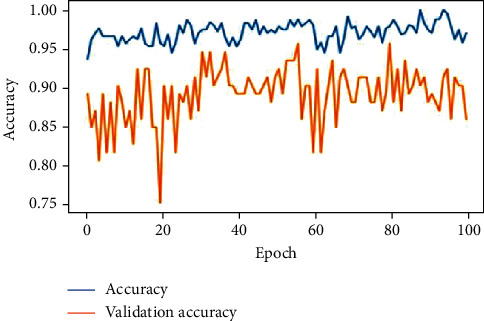
Proposed DNN after 100 epochs.

The feature extracted X-ray lung images which are subjected to the proposed deep neural network, AlexNet and VGGNet. The developed architectures classify the images into three classes of respiratory diseases as normal, COVID-19, and pneumonia. The accuracy of the lung X-ray images on test data is given in Table 7. As per the results, the proposed deep neural network was performing better when compared with the other 2 predefined networks. Figures 17–19 show the accuracy and validation accuracy per epoch which can help to determine the best model. The validation accuracy is almost on par with the model accuracy which proves that the trained model is efficient in the classification of respiratory diseases.

**Figure 20:**
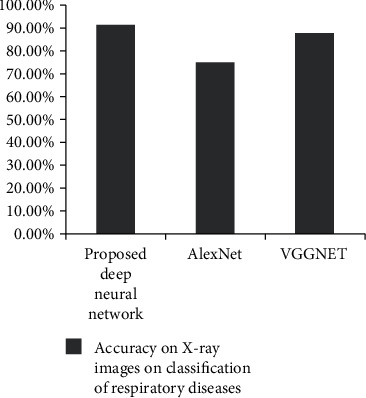
Analysis of classification accuracy.

The image preprocessing techniques shown in Figure 20 along with feature descriptors (Figure 8) applied on the dataset also aid in the classification accuracy of the proposed deep neural network model. Pseudocolor is a useful tool for contrast enhancement and visualization of data. The contrast of the X-ray images was poor, and hence to improve, the proposed method included addition of data visualization.

**Figure 21:**
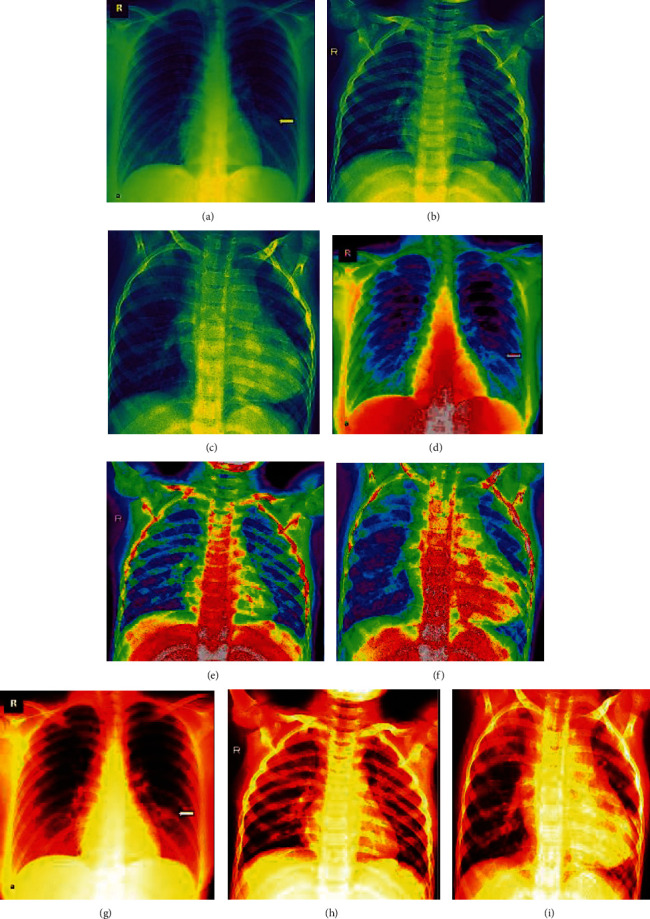
(a) Normal-colorbars; (b) COVID-19-colorbars; (c) pneumonia-colorbars. (d) Normal nipy_spectral; (e) COVID-19 nipy_spectral; (f) pneumonia nipy_spectral. (g) Normal-viridis. (h) COVID-19-viridis. (i) Pneumonia-viridis (other image preprocessing results).

The grayscale images are processes using Matplolib's pyplot. This is a powerful method suitable for large datasets. Figures 21(a)–21(c) display colorbars and indicate the data values as a colormap. The mapping highlights the most dominant features in identifying the disease. A sequential colormapping technique, Virdis (Figures 21(d)–21(f), highlights the infected portions. So, when a user refers to the developed model, he/she can be visually supported with the classification. The nipy_spectral method (Figures 21(g)–21(i)) further discriminates the anatomy of the lung with respect to normal/COVID-19 and pneumonia affected regions. The dataset is huge, and when we subject these three image processing techniques along with segmentation, feature descriptors, and finally with classification, the results are obvious and easy to predict the disease without the support of human assistance.

## 12. Discussion

The proposed method incorporated preprocessing techniques comprising noise removal and image enhancements. Segmentation and filtering were applied to identify left/right lungs. Then, the preprocessed images were subjected to classifiers for the classification of respiratory diseases. The results were compared to identify the most accurate preprocessing technique and classification model. Haar transform techniques outperformed other techniques based on the IOU scores. The proposed DNN accurately classified the diseases with 91.4% with runtime of the program to be 1944 seconds (32 minutes). The processor information is Intel® core™, 16 GB RAM, 64-bit operating system. The problem statement requires a large dataset for training the deep learning model. This is time-consuming when trained using a 32/64-bit processor. This might further reduce when simulated in GPU. Hence, based on the results, we have improved the accuracy of the classifiers by applying efficient feature extraction techniques and image preprocessing algorithms. The existing techniques on the classification of respiratory diseases are 90% accurate, and our proposed method provides an in-depth analysis of preprocessing techniques to improve the DNN model performance.

## 13. Conclusion

A novel method has been proposed for the efficient classification of respiratory diseases from chest X-ray lung images. The existing technologies focus on COVID-19 diagnosis whereas this proposed method focuses on all bacterial and viral infection lung diseases. In this pandemic situation, it is necessary to differentiate COVID-19 from pneumonia. The proposed method targets preprocessing and feature descriptors to efficiently classify life-threatening lung diseases. The chest X-ray images are preprocessed by applying various image processing algorithms. Then, the preprocessed images are subjected to Haar transform filters with various kernels to extract predominant features of the X-ray image. Gradient direction and magnitudes are calculated for every pixel, and feature vectors are also extracted using histograms. Similarly, a local binary pattern is also applied for extracting features for an image in the classification model.

Based on the features extracted by three different techniques and using an appropriate classification model, the left and right lungs are segmented.

- The features extracted through Haar transform provide the best accuracy in segmentation of left and right lungs
- Cascade classification and support vector machine aid in identifying the presence of lungs
- Segmentation of lungs using Haar cascade classifier outperforms with an average IoU score of 81% when compared with the other two algorithms
- The feature descripted X-ray images are then subjected to the proposed deep neural network, pretrained AlexNet and VGGNet for classification of lung diseases (normal/COVID-19/pneumonia)

The proposed deep neural network performed well with 91% of accuracy in classifying images into pneumonia bacteria, COVID-19, and normal. This model can help doctors to diagnose, study the diseases, and provide appropriate treatment. This architecture can be also used for diagnosing various other life-threatening diseases from medical images. Thus, the developed method focused towards achieving the major goals of the United Nations in promoting good health and well-being through diagnosis of COVID-19 and other respiratory diseases.

## Figures and Tables

**Table 1:** Dataset for building a model.

Chest X-ray image	No. of images used for training the model	No. of images used for testing the model
Normal lung	110	30
COVID-19	110	30
Pneumonia	110	30

**Table 2:** Proposed deep neural network layers.

Layer (type)	Output shape	Param #
conv2d (Conv2D)	(None, 224, 224, 64)	1792
conv2d_1 (Conv2D)	(None, 224, 224, 64)	36928
max_pooling2d (MaxPooling2D)	(None, 112, 112, 64)	0
conv2d_2 (Conv2D)	(None, 112, 112, 128)	73856
conv2d_3 (Conv2D)	(None, 112, 112, 128)	147584
max_pooling2d_1 (MaxPooling2)	(None, 56, 56, 128)	0
conv2d_4 (Conv2D)	(None, 56, 56, 256)	295168
conv2d_5 (Conv2D)	(None, 56, 56, 256)	590080
conv2d_6 (Conv2D)	(None, 56, 56, 256)	590080
max_pooling2d_2 (MaxPooling2)	(None, 28, 28, 256)	0
conv2d_7 (Conv2D)	(None, 28, 28, 512)	1180160
conv2d_8 (Conv2D)	(None, 28, 28, 512)	2359808
conv2d_9 (Conv2D)	(None, 28, 28, 512)	2359808
max_pooling2d_3 (MaxPooling2)	(None, 14, 14, 512)	0
conv2d_10 (Conv2D)	(None, 14, 14, 512)	2359808
conv2d_11 (Conv2D)	(None, 14, 14, 512)	2359808
conv2d_12 (Conv2D)	(None, 14, 14, 512)	2359808
max_pooling2d_4 (MaxPooling2)	(None, 7, 7, 512)	0
flatten (flatten)	(None, 25088)	0
dense (dense)	(None, 4096)	102764544
dense_1 (dense)	(None, 4096)	16781312
dense_2 (dense)	(None, 3)	12291
Total params: 134272835; trainable params: 134272835; nontrainable params: 0		

**Table 3:** AlexNet: deep neural network layers.

Layer (type)	Output shape	Parameter #
conv2d_13 (Conv2D)	(None, 54, 54, 96)	34944
activation (activation)	(None, 54, 54, 96)	0
max_pooling2d_5 (MaxPooling2)	(None, 27, 27, 96)	0
conv2d_14 (Conv2D)	(None, 17, 17, 256)	2973952
activation_1 (activation)	(None, 17, 17, 256)	0
max_pooling2d_6 (MaxPooling2)	(None, 8, 8, 256)	0
conv2d_15 (Conv2D)	(None, 6, 6, 384)	885120
activation_2 (activation)	(None, 6, 6, 384)	0
conv2d_16 (Conv2D)	(None, 4, 4, 384)	1327488
activation_3 (activation)	(None, 4, 4, 384)	0
conv2d_17 (Conv2D)	(None, 2, 2, 256)	884992
activation_4 (activation)	(None, 2, 2, 256)	0
max_pooling2d_7 (MaxPooling2)	(None, 1, 1, 256)	0
flatten_1 (flatten)	(None, 256)	0
dense_3 (dense)	(None, 4096)	1052672
activation_5 (activation)	(None, 4096)	0
dropout (dropout)	(None, 4096)	0
dense_4 (dense)	(None, 4096)	16781312
activation_6 (activation)	(None, 4096)	0
dropout_1 (dropout)	(None, 4096)	0
dense_5 (dense)	(None, 1000)	4097000
activation_7 (activation)	(None, 1000)	0
dropout_2 (dropout)	(None, 1000)	0
dense_6 (dense)	(None, 3)	3003
Total params: 28040483; trainable parameters: 28040483; nontrainable parameters: 0		

**Table 4:** VGGNet: deep neural network layers.

Layer (type)	Output shape	Parameter #
conv2d (Conv2D)	(None, 444, 444, 32)	2432
max_pooling2d (MaxPooling2D)	(None, 222, 222, 32)	0
conv2d_1 (Conv2D)	(None, 220, 220, 64)	18496
max_pooling2d_1 (MaxPooling2)	(None, 110, 110, 64)	0
dropout (dropout)	(None, 110, 110, 64)	0
conv2d_2 (Conv2D)	(None, 108, 108, 128)	73856
max_pooling2d_2 (MaxPooling2)	(None, 54, 54, 128)	0
dropout_1 (Dropout)	(None, 54, 54, 128)	0
conv2d_3 (Conv2D)	(None, 52, 52, 512)	590336
max_pooling2d_3 (MaxPooling2)	(None, 26, 26, 512)	0
dropout_2 (Dropout)	(None, 26, 26, 512)	0
conv2d_4 (Conv2D)	(None, 24, 24, 512)	2359808
conv2d_5 (Conv2D)	(None, 22, 22, 128)	589952
conv2d_6 (Conv2D)	(None, 20, 20, 64)	73792
max_pooling2d_4 (MaxPooling2)	(None, 10, 10, 64)	0
dropout_3 (dropout)	(None, 10, 10, 64)	0
flatten (flatten)	(None, 6400)	0
dense (dense)	(None, 4096)	26218496
dense_1 (dense)	(None, 1024)	4195328
dropout_4 (dropout)	(None, 1024)	0
dense_2 (dense)	(None, 3)	3075
Total parameters: 34125571; trainable parameters: 34125571; nontrainable parameters: 0		

**Table 5:** Existing methods on classification models.

Author Ozturk et al.	Methodology Darknet model	Year of publication 2020	Accuracy on multiclass (%) 87.02%
Civit-Masot et al.	VGG-16	2020	90%
Cohen et al.	Regression model	2020	1.14 mean absolute error

**Table 6:** Intersection over union scores for detected left and right lungs.

Algorithm Hog feature descriptor	Right lung (IOU) 0.59	Left lung (IOU) 0.65
Haar transform	0.86	0.81
Local binary pattern	0.36	0.54

**Table 7:** Performance of deep learning models.

Deep learning models Proposed deep neural network	Accuracy on X-ray images on classification of diseases 91.40%
AlexNet	75.00%
VGGNet	87.5%

## Data Availability

The data used to support the findings of this study are included within the article.
